# Probiotics and Colon Cancer

**DOI:** 10.3390/microorganisms7030066

**Published:** 2019-02-28

**Authors:** Lorenzo Drago

**Affiliations:** Clinical Microbiology, University of Milan, 20100 Milan, Italy; lorenzo.drago@unimi.it

**Keywords:** colon cancer, gut microbiota, probiotics, anticancer effect

## Abstract

Literature has recently highlighted the enormous scientific interest on the relationship between the gut microbiota and colon cancer, and how the use of some selected probiotics can have a future impact on the adverse events which occur during this disease. Although there is no clear evidence to claim that probiotics are effective in people with cancer, recent reviews have found that probiotics can significantly reduce the incidence of diarrhea and the average frequency of daily bowel movements. However, most of this evidence needs to be more clinically convincing and further discussed. Undoubtedly, some probiotics, when properly dosed and administered, can have a strong rebalance effect on the gut microbiota and as a consequence a possible positive action on immune modulation of the gastrointestinal tract and on inflammation of the intestinal mucosa. Many recent findings indeed support the hypothesis that the daily use of some selected probiotics can be a feasible approach to effectively protect patients against the risk of some severe consequences due to radiation therapy or chemotherapy. This paper aims to review the most recent articles in order to consider a possible adjuvant approach for the use of certain well-balanced probiotics to help prevent colon cancer and the adverse effects caused by related therapies.

## 1. Introduction

An ever-increasing literature demonstrates that the microbiota can have a strong influence on colon cancer (CC) prevention and on the healing process during an intestinal inflammation. Understanding the mechanisms by which the bacterial community of the gut influences intestinal diseases could pave the way for new preventive and therapeutic approaches. Several mechanisms were discovered in recent years, the most evident one was the influence of the production of specific reactive oxygen species (ROS) and the activation of specific peptide receptors in regulating intestinal homeostasis. Very recent evidence suggests that specific gut microbes, such as *Lactobacillus* spp. can help to positively regulate these processes [1].

Thanks to the advent of Next Generation Sequencing methodologies, it is possible to define the gut microbiota as a complex community of microbes that number over 10^14^ cells, consisting of bacteria, fungi, protozoa, viruses, and bacteriophages, which reside within the gut and live in a symbiotic and epigenetic relationship with the host.

**Figure 1:**
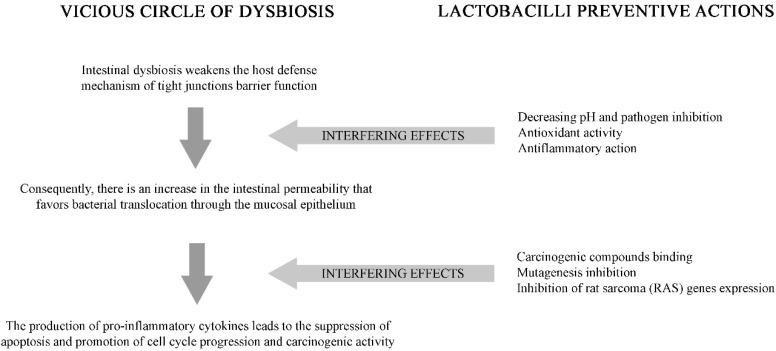
Microbiota and colon cancer: The vicious cycle of dysbiosis activity of selected Lactobacilli.

Indeed, it is widely demonstrated that epigenetic changes and gene regulations can also occur during the development of colon cancer (CC). Along with factors such as diet, lifestyle, genetics, and oncogenic infection, specific microorganisms or the variability of the microbiome, have been recently associated with this tumor. How gut microbiome contributes to CC pathogenesis in the host is not fully understood. The gut microbiota associated with CC reveals a dynamic and complex microbial interaction, which is under strong consideration by scientists who want to study the mechanisms related to the development of CC. During this multifactorial carcinogenic process, a gradual alteration of microbiota, along with their microenvironment which causes dysbiosis and increases potential oncopathogenic microbes, can mediate the modulation of cancer (Figure 1). Undoubtedly, colon tumorigenesis is also related to the role of some microbial metabolites as an initiator or inhibitor of procarcinogenic or antioncogenic activities [2].

*Fusobacterium nucleatum* and *Porphyromonas gingivalis* are identified as cancerogenic bacteria. Their overabundance of sequences in tumors versus matched normal control tissue, and their positive association with lymph node metastasis has been observed [3,4,5]. All these studies suggest that some microorganisms can represent a novel risk factor for disease progression from adenoma to cancer, possibly affecting patient survival outcomes. Looking at this scenario, it could be strategically relevant to counteract the negative outcomes due to the presence of these microorganisms by using some selective bacteria with inhibitory effects against the pathobionts.

For future perspectives, the evaluation of the microbiome in the development of new markers and therapeutic agents in CC is highly recommended.

The list of health-promoting effects attributed to probiotic bacteria is extensive and includes the alleviation of the symptoms of lactose intolerance, serum cholesterol reduction, anticancer effects, the improvement of constipation/diarrhea, and the relief of vaginitis. The vast majority of studies on anticancer effects deal with colorectal cancer, although others are related to breast and bladder cancer [6].

Classically, the definition of probiotics is “live microorganisms which, when administered in adequate amounts, confer a health benefit to the host” [7]. Interestingly, bacteria belonging to *Lactobacillus* and *Bifidobacterium* genera are the most used probiotic microorganisms in the food industry, due to their probiotic and beneficial effects. Recently, the “10 commandments or recommendations”, an instruction kit for physicians to follow and to give an easy and immediate interpretation of the probiotic(s) under consideration, has been published [8].

The present review will discuss the most recent knowledge and future perspectives concerning the potential use of specific probiotics in CC. Recently available evidence starting from animal studies to human conditions, as well as the use of probiotics for the prevention or therapy of CC, and the related adverse events, will be also addressed. 

## 2. Colon Cancer and Probiotics in an Animal Model

Inflammatory and carcinogenic stimuli cause changes in the composition of the gut microbiota that may predispose to tumorigenesis. In a study by Zackular et al. [9] the treatment of mice with carcinogen azoxymethane (AOM), followed by the inflammatory compound dextran sulfate sodium (DSS), was associated with dramatic alterations in the microbial community and significant changes in relative microbial abundances. In addition, germ-free mice that were recolonized with the gut microbiota of tumor-bearing mice developed more tumors compared with those harboring the microbiota of naïve healthy mice after treatment with AOM/DSS. This study demonstrated that these changes directly contributed to tumor susceptibility and the alteration of the intestinal microbiota was an important determinant of colon tumorigenesis. 

Although this mechanism of protection has been well demonstrated, other articles have suggested the role of specific commensal bacteria in limiting inflammation-associated colon tumorigenesis by activating several pathways, which are not definitively understood thus far [10,11].

Bacterial metabolites have also been seen to have a protective effect. Butyrate for instance, which can only be produced by specific members of the *Firmicutes* phylum through the fermentation of dietary fiber and resistant starches, can modulate inflammation, epithelial proliferation, and apoptosis [12]. Ganapathy et al. [13] gave evidence that butyrate was recognized by the host colonic receptors GPR109 and GPR43, and mice who were deficient in GPR109 exhibited increased tumor susceptibility and colon tumorigenesis in two different colon cancer mouse models. This evidence was also demonstrated in a study by Singh et al. through the butyrate activation of the GR109a receptor [14].

On the other side, Belcheva et al. [15] demonstrated that butyrate may instead promote epithelial proliferation, and as a consequence, increase susceptibility to tumorigenesis in the case of genetic modifications. In this mouse model, which harbored a mutation in the APC (adenomatous polyposis coli) tumor suppressor gene, butyrate stimulated colon epithelial proliferation and increased the number of tumors. It has been seen in mice harboring an APC mutation in the intestinal epithelium, that adenoma formation is associated with the upregulation of cytokines, such as IL-6, IL-17, and IL-23 and the induction of proinflammatory Th17 cells [16].

Of course, other microbial metabolites can also be involved in colon tumorigenesis. In a recent animal colorectal cancer (CRC) study [17], scientists illustrated the ways to replace missing metabolites in patients prone to gut inflammation and CRC, and how specific probiotics may become a new preventative or therapeutic strategy for patients at risk for inflammatory bowel disease (IBD)-associated CRC. This study, published in The American Journal of Pathology, described how the administration of histamine-producing gut microbes to mice lacking the enzyme histidine decarboxylase (HDC) can reduce inflammation and tumor formation. 

Other studies emphasized the specific role of Lactobacilli in preventing colon cancer in the animal model [18,19,20]. These studies revealed the protective effects of *Lactobacillus acidophilus* and *Lactobacillus salivarius* on the development of precancerous growths and colorectal carcinogenesis in the rat model, respectively. Another study [21] showed how a specific strain of *Lactobacillus plantarum* inhibited colon cancer in the mouse model after the chemical induction of cancer. 

Some authors reported that lower intracolonic pH values inhibited the proliferation, and consequently the activity, of putrefactive carcinogenic bacterial enzymes. Thus, Chang et al. [18] attributed the reductions in the intestinal populations of carcinogenic bacteria to the low intracolonic pH exhibited by the rats treated with *L. acidophilus* at a high dosage for 10 weeks. 

All these studies, taken together, represent how specific strains of probiotics can interfere with external conditions, such as the inflammatory process, dysbiosis, or aberrant colon metabolism, leading to tumorigenesis. 

## 3. Microorganisms Responsible for or Protective of Colon Cancer

Nowadays it is not easy to identify a particular bacterial population or change in their abundance or number of single strains responsible for increasing tumor susceptibility and development. Studies have identified that several bacteria, not only *Fusobaterium nucleatum* or *P. gingivalis* as mentioned before, but also *Escherichia coli*, *Bacteroides fragilis*, and *Enterococcus faecalis*, were increased in CC patients, whereas the *Clostridiales*, *Faecalibacterium*, *Blautia*, *Bifidobacterium*, or *Lactobacillus* genus were absent [22,23,24]. Of course, particular populations or their abundance cannot be definitively related to tumorigenesis. Clostridia, for instance, are often identified as oncomicrobes [11,25], but the complexity of microbiota and the relationship between microorganisms (network) in the gut does not underline a particular bacterial signature for tumor progression so far. Undoubtedly, the gut microbiota can promote either health or tumor progression (in case of dysbiosis for instance) through its inflammatory and proliferative effects likely dependent on the context and genetic factors of the host as well.

Among many available probiotic strains widely used clinically, specific Lactobacilli (but also Bifidobacteria) are well characterized in terms of their activity and anti-inflammatory role in modulating cytokine production in human dendritic cells [26,27] (Figure 1). A study by Kuugbee et al. [28] demonstrated that *Lactobacillus* administration to mice regulated the expression of Toll-like receptor 2 (TLR2), TLR4, and TLR9, especially TLR2, and decreased tumor incidence.

Cancer is one of the scenarios where too little apoptosis occurs, resulting in malignant cells that will not die. The mechanism of apoptosis is complex and involves many pathways. Gamallat et al. [29] demonstrated that a specific strain of *L. rhamnosus* as a prophylactic measure could reduce the incidence and multiplicity of colon tumors by inducing cell apoptosis and inhibiting inflammation, while Ciorba et al. [30] evidenced the activity of specific Lactobacilli in enhancing the intestinal epithelial barrier function in a TLR2/cyclo-oxygenase-2-dependent manner. Ghoneum and Felo [31] reported the effect of *Lactobacillus kefiri*, a novel kefir product, on the rate of apoptosis of gastric cancer cells, breast cancer cells, and human peripheral blood mononuclear cells (PBMCs). The results evidenced a potential therapeutic agent for the treatment of gastric cancers. 

Free radicals are highly reactive chemicals harmful for the cells in developing the tumor. A recent study emphasized the importance of selecting Lactic acid bacteria as the progenitor strain due to its innate beneficial properties in producing anti-oxidant enzymes. This study showed the best anti-cancer effect of mixtures of Lactobacilli by combining different anti-inflammatory mechanisms and IL-10 stimulation in a colorectal cancer mouse model [32].

The study by Verna et al. [33] showed that the suppression of some oncogenic enzymes can be modulated by the oral intake of selected probiotics. This study demonstrated that the combination of *L. rhamnosus* and *L. acidophilus* also suppressed the preneoplastic aberrant crypt foci in a rat animal model. 

Some strains of probiotics are able to metabolize and inactivate specific compounds, such as the N-nitroso compounds and heterocyclic aromatic amines [34]. All these activities, and especially the degradation of carcinogenetic enzymes, appear to be dependent on the strain and viability used as probiotics, as well as on the specific host condition (environmental intestinal conditions, such as pH, the presence of bile salts, and gastrointestinal enzymes). 

Several species of probiotic bacteria, such as *L. acidophilus*, *L. casei*, *L. plantarum*, *Propionibacterium freudenreichii*, *Lactobacillus delbrueckii*, *Bifidobacterium infantis*, *Bifidobacterium breve*, *Bifidobacterium longum*, and *Streptococcus thermophilus* may also produce conjugated linoleic acids from linoleic acid. The fatty acids produced by these bacteria can act into the colonocytes by exerting antiproliferative and proapoptotic activities with locally beneficial effects [35]. 

## 4. Probiotics in Colon Cancer Prevention and Treatment

Natural sources which confer anti-carcinogenic effects for the prevention of colon cancer, such as probiotics, have been receiving important focus in recent years [36].

Several studies have suggested that regular consumption of probiotics may improve the quantitative and qualitative profile of the intestinal microbiota, thus reducing the trigger of chronic inflammation and the production of carcinogenic compounds during intestinal dysbiosis [37,38,39]. In a study by Liu et al. [39] the regular consumption of *L. plantarum*, *L. acidophilus*, and *B. longum* at a high dosage for 16 days, increased the diversity and microbial richness in individuals with CRC undergoing a colorectomy. In this study, the intestinal microbiota composition of patients resembled that of the healthy individuals. As mentioned, some intestinal enzymes, such as β-glucosidase, β-glucuronidase, nitrate reductase, azoredutase, and 7-α-dehydroxylase, which are able to convert aromatic hydrocarbons and amines in active carcinogens by synthetizing aglycones, phenols, cresols, ammonia, and N-nitroso compounds, can have cytotoxic and genotoxic activities, thereby contributing to the development of colon cancer. The vivo study by Hatakka et al. [37] demonstrated that the consumption of certain strains of probiotic bacteria can reduce the activity of these enzymes and prevent colon cancer. Certain probiotics can also influence the immune response through the activation of phagocytes, and contribute to the maintenance of the state of immune-vigilance, which can eliminate cancer cells in their early stages of development [40,41]. It is worthy to note that the immunomodulatory properties are strain dependent; the survival and the persistence into the gastrointestinal tract, as well as the posology, can also strongly influence the immune system. Therefore, not all probiotics are able to modulate the immune system and to prevent the occurrence of CC. Interestingly, Galdeano et al. [41] highlighted the importance of the dosage (around 10^9^ colony forming unit-CFU/day) and the intestinal permanence time, which ranged between 48 and 72 h, as the optimal characteristics to induce immunostimulation on the host. 

One promising application for the treatment of colorectal cancer is the human microbiota manipulation, and as a consequence, the use of selective probiotics. To my knowledge there are few preliminary studies, especially randomized and controlled, that evaluate whether manipulation of the microbiota in patients receiving treatment for colorectal cancer may affect outcomes, such as the objective response rate or progression-free survival. 

Nevertheless, it has been widely demonstrated that the regular consumption of probiotics can reduce intestinal permeability by changing the distribution of cell junction proteins [42,43] and decreasing the amount of potential carcinogenic compounds absorbed and acting negatively on the colonocytes. Treatment with a mixture of probiotics (*L. plantarum*, *L. acidophilus* and *B. longum*) in individuals with CRC improved the outcome and increased the amount of cell junction proteins (claudin, occludin, and JAM-1) as well as their distribution throughout the colonic epithelium [39]. 

The proapoptotic activity induced by the consumption of probiotics, especially by increasing TNF-α production, is another factor well investigated in human cancer [44]. Wan et al. [45] concluded that the ability to induce apoptosis of the tumor cells by the probiotic *L. delbrueckii* was a consequence of the increased expression of caspase-3 inducing aptoptosis. 

In a randomized control trial with colon cancer and polypectomized patients, Rafter et al. [46] demonstrated a reduction of several cancer biomarkers and a decreased genotoxic exposure (IL-2 and INF-gamma) after oral treatment with *L. rhamnosus* and *B. breve* combination. 

Recently, Kotzampassi et al. [47] observed a significant decrease in all major post-operative complications in patients undergoing surgery for colon rectal cancer after treatment with a probiotic formulation versus a placebo (28.6% vs. 48.8%, *p* = 0.010, OR 0.42). All these studies are too far for a clear demonstration that probiotics can prevent or treat colon cancer, but they could represent a promising start for further investigations in this intriguing field. In these studies, potential confounding factors such as BMI, smoking, type of diet, and physical activity can also be accounted for. An Italian prospective cohort study showed that self-reported yogurt intake had an inverse association with colorectal cancer risk, but the authors evidenced that the above-mentioned factors can influence this result [48].

A very recent and fascinating field, the manipulation of the microbiota composition during CC immunotherapy may lead to the development of alternative therapeutic strategies in colon cancer. These very preliminary researches are revealing an important interplay between intestinal microbiota and the immune system, and include the possibility of targeting the microbiota for the enhancement of anticancer treatment. Of course, additional and deeper studies will be necessary to evaluate the interaction between the microbiota and colon cancer immunotherapy [49]. Recently, it has become evident that microbiota, particularly the gut microbiota, can modulate the response to cancer therapy and the susceptibility to toxic side effects. The evidence demonstrating the ability of the microbiota to modulate chemotherapy, radiotherapy, and immunotherapy with a particular focus on microbial composition, has started to become very impressive [50,51].

## 5. Probiotics as Adjuvant in Adverse Events

The use of probiotics as adjuvant to improve the safety and gastrointestinal side effects during cancer treatment has also been explored by evaluating clinically the possible benefit of probiotics during and after surgery, radiation therapy, and chemotherapy. In this regard, probiotics are very attractive as a potential complement in these circumstances because they are inexpensive if we consider the sanitary costs for the cancer management [52,53,54].

The primary reasons that probiotics may be beneficial in mitigating the adverse gastrointestinal effects of cancer treatment are demonstrated in many animal models. Mice intraperitoneally-injected with 5-Fluorouracil (5-FU) developed diarrhea, but their symptoms were alleviated after treatment with a probiotic suspension. The study also demonstrated the mechanisms for which it occurs: Repairing damages in the jejunal villi, and reducing mRNA expression of TNF-alpha, IL-1 beta, and IL-6 in intestinal tissue [55]. Another study [56] underlined the activity of a mixture of different microorganisms in reducing the severity of diarrhea and improving histological examination in a mouse experimental model. 

In human cancer, some published data discusses whether probiotics can help to improve tolerance to chemotherapy. In a recent randomized control trial, colorectal cancer patients starting treatment with irinotecan were concurrently treated orally with a mixture of probiotic bacteria. Although the study was interrupted, the trend observed was in favor of reducing the frequency of severe diarrhea [54].

Another clinical trial [57] demonstrated that the supplementation *L. rhamnosus* can reduce the severity of diarrhea, abdominal discomfort, and bowel toxicity in patients under treatment with 5-FU-based chemotherapy regimens. 

Probiotics can also be useful in the setting of radiation therapy. A meta-analysis published many years ago by Fuccio et al. [58], which considered only four studies, demonstrated a significant positive effect in a few, but not an overall benefit when the studies were considered all together. Other studies have demonstrated that probiotics can improve and repair radiation-induced injuries [59] as well as the incidence and severity of radiation-induced diarrhea, the daily number of bowel movements, and the time from the start of the study to the use of loperamide as a rescue medication [53].

As mentioned before, literature reports also provide results on trials evaluating probiotics as an adjunctive treatment to the colon cancer surgery [47,52].

Although these studies have given some positive feedback, only more in-depth and detailed scientific approaches can give a definitive response for using probiotics as adjunctive therapy for a better outcome against the detrimental effects of anticancer therapies. Of course, not all the probiotics are useful or the same, and this could explain how the variation of probiotic strains, doses, and regimens between the different studies can deeply influence the results.

## 6. Conclusions

Understanding factors influencing the gut microbiome, strategies to augment therapeutic responses, as well as engineering the microbiome for precision therapy as future perspectives are mandatory. Dietary interventions and food supplements, such as some selected probiotics, have emerged as a valid alternative to manage colon cancer. It is also relevant that very few reports demonstrate any adverse effects of probiotic oral supplementation. However, this is another aspect which needs to be further clarified, especially in the immunocompromised hosts who usually consume probiotics as a diet supplement. We clearly know that some not well-characterized probiotics can alter the intestinal barrier. This is the main reason why it will be necessary in the future to follow strict rules and have an efficient selection of bacteria to be included in the “safe and effective probiotics list”. Genetic identification, toxicity, gastrointestinal resistance, and colonization rate are the main requisites to design a good probiotic [8]. 

Recent studies have identified overrepresentation of *Fusobacterium* in colorectal cancer tissues, but it is not yet clear whether this is pathogenic or simply an epiphenomenon. Findings show that *Fusobacterium* enrichment is associated with specific molecular subsets of colorectal cancers, offering support for a pathogenic role in colorectal cancer for a gut microbiome component [60,61]. Lactic acid bacteria bacteriocins and probiotic preparations could provide an alternative for antibiotics [62]. Some selected probiotics by producing bacteriocins, such as lantionine (Lan), methyllantionine (MeLan), dehydroalanine (Dha), dehydrobutyrine (Dhb), or D-alanine (D-Ala), could have an inhibitory effect against the overgrowth of the above-mentioned microorganisms. 

Contradictory results still exist in literature about the anticarcinogenic activities of probiotics. Again, these discrepancies can be explained by the fact that the mechanisms are strain specific. Therefore, further studies should be conducted to identify which specific strains are involved in the prevention or treatment of CC. It is never enough to say that the studies reported in literature used varying doses, treatment times, and frequency use, as well as delivery of probiotic product (lyophilized, microencapsulated, or yogurt). These aspects can lead to different results and make it challenging to compare the results from such studies. Another important aspect that could minimize the efficacy of orally administrated probiotics is the loss of viability of probiotics reaching the colon. Thus, developing and applying techniques to ensure the viability of the probiotics strains, such as microencapsulation, could enhance the preventive effect of such strains on the CC development. 

In conclusion, many scientific evidences suggest that the consumption of some selected probiotics can contribute to the prevention of colon cancer by exerting anticarcinogenic activity by potential physiological mechanisms that are usually host-dependent and strain-specific. Recent findings also suggest that some probiotics can alleviate adverse symptoms related to anticancer therapy. Table 1 summarizes the 10 basic mechanisms by which some selected probiotics can exert their anticancer activity.

Indeed, some very interesting and well-tailored studies clearly demonstrated these activities, especially in the animal models. However, more studies are needed to elucidate which mechanisms are effective in humans. Although promising results are coming from the studies on the interaction of microbiome-colon cancer, data evaluating probiotics as a potential means to affect cancer are still too scarce to formulate a definitive response. Future perspectives that allow a fuller description of gut biodiversity and accurately monitor changes in response to anticancer therapy, will help to determine the role of probiotics in cancer prevention and in alleviating the adverse effects occurring consequently. This research should pave the way in the future for “the era of anticancer probiotics” aimed at restoring gut eubiosis for a better clinical outcome in cancer patients.

## Figures and Tables

**Table 1:** The 10 golden mechanisms of some selected probiotics strains in colon cancer (CC).

Effect	Model Study	Listed Probiotics in CC Studies
Promotion of epithelial repair and barrier	Animal/Human [17,29,34,35,42,43]	*Lactobacillus acidophilus Lactobacillus salivarius Lactobacillus plantarum Lactobacillus rhamnosus Lactobacillus kefiri Lactobacillus casei Lactobacillus delbrueckii Bifidobacterium infantis Bifidobacterium breve Bifidobacterium longum Streptococcus thermophilus*
Negative regulation of inflammatory pathways promoting tumorigenesis	Animal/Human [9,10,27,28,33,36,41,42]	
Increased tumor cells apoptosis	Animal/Human [12,29,31,45]	
Prevention of dysbiosis and restoring eubiosis	Animal/Human [8,38,39,40,50]	
Upregulation of cytokines promoting tissue repair and antitumor responses	Animal/Human [16,44,46]	
Production of metabolites with positive effects on the epithelium and immune cells (SCFAs, acetate, propionate, butyrate)	Animal/Human [13,14,17,37]	
Selective exclusion of pathogenic and tumorigenic bacteria (bacteriocines)	Animal/Human [18,39,62]	
Inhibition of Biofilm formation and thus cell proliferation via Toll-like receptors	In vitro/Animal [28,62]	
Improvement of adverse events during chemotherapy and radiation therapy	Human [53,55,56,57,58,59]	
Synergistic effect with anti-cancer and immunological drugs for improving their kinetics properties	Hypothesis/Studies in progress [50,51]	
